# Characterization, complete genome sequencing, and CRISPR/Cas9 system-based decontamination of a novel *Escherichia coli* phage TR1 from fermentation substrates

**DOI:** 10.3389/fmicb.2023.1230775

**Published:** 2023-08-11

**Authors:** Yuqi Dong, Yunfei Huang, Huahao Fan, Lihua Song, Xiaoping An, Shan Xu, Mengzhe Li, Yigang Tong

**Affiliations:** ^1^College of Life Science and Technology, Beijing University of Chemical Technology, Beijing, China; ^2^Qinhuangdao Bohai Biological Research Institute, Beijing University of Chemical Technology, Qinhuangdao, Hebei, China; ^3^Beijing Advanced Innovation Center for Soft Matter Science and Engineering, Beijing University of Chemical Technology, Beijing, China

**Keywords:** fermentation, phage contamination, *Escherichia coli*, CRISPR/Cas9, defense system

## Abstract

Phage contamination has become a major concern for industrial bacteria, such as *Escherichia coli* BL21(DE3), used in fermentation processes. Herein, we report a CRISPR/Cas9 defense system-based strategy to precisely prey and degrade phage DNA to decontaminate target phages. First, we isolated a novel phage from fermentation substrates with BL21(DE3) as the host, named TR1. It showed a typical podovirus morphology with a head diameter of 51.46 ± 2.04 nm and a tail length of 9.31 ± 2.77 nm. The burst size of phage TR1 was 151 PFU/cell, suggesting its strong fecundity in the fermentation system. Additionally, whole-genome sequencing revealed that phage TR1 has a DNA genome of 44,099 bp in length with a 43.8% GC content, encoding a total of 68 open reading frames. Comparative genomics and phylogenetic analysis designated this phage to be a new species of the genus *Christensenvirus*. To counteract phage TR1, we employed the CRISPR/Cas9 system-based strategy and constructed two phage-resistant *E. coli* strains, BL21-C and BL21-T, based on conserved genes. Both EOP assays and growth curves indicated strong phage resistance of the recombinant strains, without affecting cell growth. Therefore, this study aimed to provide a resilient strategy to respond to ever-changing phages and ongoing phage–host arm race in industrial fermentation environments by the personalized design of spacers in the recombinant CRISPR/Cas system-containing plasmid. More importantly, our research sparks the use of phage defense mechanism to prevent phage contamination in extensive biotechnological applications.

## Introduction

*Escherichia coli* (*E. coli*) BL21(DE3), a Gram-negative bacteria, has long been a model organism in cell factories, a mainstay of many biotechnological applications such as recombinant protein production and chemicals used in fuels, materials, and medicine ingredients field (Nielsen and Keasling, 2016; Kim et al., 2017). However, phage contamination has been a tough issue in the fermentation industry (Baltz, 2018), leading to the destruction of bacterial cultures that results in fermentation failure, paralysis of facility productivity, and consequently heavy financial losses (Los, 2012). Physical treatments, such as ionizing radiation, thermal treatments, and high pressure, can eliminate some phages but may be ineffective against others (Guglielmotti et al., 2011). Currently, many disinfectants used in the industry have a limited ability to reduce the phage population because not all sanitizers and biocides are effective against phages, with various phages showing varied susceptibility to a given disinfectant (Guglielmotti et al., 2011; Los, 2012). Therefore, it is important to explore a more widely effective strategy to prevent phage contamination.

For hundreds of years, bacteria have developed an amazing array of strategies to fight these phages at each step of the infection process during the co-evolution of bacteria and phages, typically carrying a wide repertoire of defense systems to prevent phage lysis (Labrie et al., 2010; Makarova et al., 2013; Rocha and Bikard, 2022). The initial step of infection is phage adsorption to bacteria by recognizing a specific bacterial component such as lipopolysaccharide (LPS), the outer membrane protein (Bertozzi Silva et al., 2016), and flagella. Accordingly, bacteria have developed mechanisms to prevent this process, such as receptor blocking, competitive inhibitor production, and extracellular matrix production (Labrie et al., 2010). No matter which strategy we choose to remold engineering strains to resist phages, phages can evolve resistance mechanisms correspondingly during the phage–host arms race (Azam and Tanji, 2019). After phage adsorption, phage DNA is injected into bacterial cells and the replication and transcription are carried out subsequently. During this process, bacteria have evolved to counteract phages, including superinfection exclusion (Sie) systems (Bondy-Denomy et al., 2016), restriction-modification (RM) systems (Roberts et al., 2003), bacteriophage exclusion (BREX) systems (Goldfarb et al., 2015), abortive infection (Abi) systems (Lopatina et al., 2020), defense island system associated with restriction-modification (DISARM) systems (Ofir et al., 2018), and toxin and anti-toxin (TA) systems (LeRoux and Laub, 2022). Among all these defense systems, clustered regularly interspaced short palindromic repeats-CRISPR-associated protein (CRISPR/Cas) system is generally used as a tool for genome engineering (Hryhorowicz et al., 2017). It is worth noting that the original function of CRISPR/Cas systems is to provide resistance against the foreign DNA from phage invasion (Safari et al., 2020), which is a natural tool to fight against phage invasion. For example, CRISPR/Cas9, a typical member of the type II-A CRISPR system (Makarova and Koonin, 2015), was discovered in *Streptococcus pyogenes* bacteria as an adaptive immune system (Yuen et al., 2015), protecting bacteria and archaea against foreign genetic elements such as phages or plasmids. The system recognizes the foreign DNA as spacers by generating crRNAs. When crRNAs combine with tracrRNA, the complex that is called single guide RNA (sgRNA) guides the Cas9 protein to the foreign DNA sequence (Hynes et al., 2016). Once the Cas9 protein binds to the sequence, the target DNA becomes degraded or inactivated (Makarova and Koonin, 2015; Hynes et al., 2016). Subsequently, when the same foreign sequence invades bacterial cells, it can be recognized and neutralized quickly by sgRNA (Ma et al., 2014). In view of this process, the spacers of the CRISPR/Cas9 system can be designed according to phage genome sequences to escape phage invasion.

In this study, we isolated a novel phage named TR1 that contaminated the fermentation substrates of *E.coli* BL21(DE3) for protein production in the Chinese Academy of Sciences. To solve the phage contamination during the fermentation, we analyzed the biological characterization and genomic feature of phage TR1 and then provided a strategy for conferring resistance to phages on the bacteria by introducing a defense element based on CRISPR/Cas9 system.

## Materials and methods

### Bacteria culture conditions

*E. coli* BL21(DE3) was incubated in Luria-Bertani (LB) broth at 37°C with shaking. In addition, a total of 25 bacterial strains, including *E. coli, Acinetobacter baumannii* (*A. baumannii*)*, Pseudomonas aeruginosa* (*P. aeruginosa*)*, Salmonella*, and *Stenotrophomonas maltophilia* (*S. maltophilia*), were used in the host range assay, and their information is provided in Table 1. The plasmid pTCPLS, which contains a CRISPR/Cas9 system, was kindly provided by Dr. Shuai Le from the Chinese Third Military Medical University of the People's Liberation Army. The plasmid carries a gentamycin acetyltransferase gene that confers resistance to gentamicin. In order to induce the activity of the Cas9 protein, recombinant bacteria harboring the plasmid were grown in LB broth supplemented with 0.2% arabinose. Additionally, gentamicin was added at a final concentration of 20 μg/mL as a selective agent for the plasmid.

**Table 1:** Host range analysis of phage TR1 against 25 strains.

**Strain**	**Infectivity**	**Species**	**Description**
BL21(DE3)	+		
2517	+		BL21(DE3) mutants of *ΔompF* deletion
2518	+		BL21(DE3) mutants of *LPS* deletion
2519	+		BL21(DE3) mutants of *ΔTsx* deletion
2520	+		BL21(DE3) mutants of *ΔompA* deletion
2523	+		BL21(DE3) mutants of *ΔFadL* deletion
MG1655	-		
DH-5α	-		
ATCC25922	-	*Escherichia coli*	
ATCC15597	-		
4159	-		Clinical strains of Qingyang People's Hospital
4180	-		
4320	-		
4347	-		
4363	-		
4394	-		
4411	-		
3835	-	*Acinetobacter baumannii*	Clinical strains of Affiliated Hospital of Qingdao University
3837	-		
BPA-8	-	*Pseudomonas aeruginosa*	Clinical strains of Army Medical University
BPA-10	-		
CMCC50001	-	*Salmonella*	
ATCC14028	-		
3757	-	*Stenotrophomonas maltophilia*	Clinical strains of China Medical University Aviation General Hospital
3759	-		

### Phage isolation and preparation

We used the host BL21(DE3) as the “bait” to isolate phages from the contaminated fermentation substrates in the laboratory of the Chinese Academy of Sciences (117°15′38.20″E, 31°51′4.32″N) *via* the method described by Han et al. (2022). Briefly, 1 mL of the fermentation substrates were centrifuged at 8,000 rpm for 2 min, and then the supernatant was filtered with 0.22 μm filters (JINTENG Ltd., Tianjin, China) to discard the remaining bacteria. Afterward, 100 μL of the serial 10-fold Diluted resulting solution and 200 μl of overnight BL21(DE3) culture were mixed in 5 mL of LB soft agar, and the mixture was plated on a bottom LB agar. After incubation at 37°C for 12 h for phage plaque formation, the single phage plaque was picked up and inoculated into 1 mL of phosphate-buffered saline buffer. The abovementioned steps were repeated at least three times and finally phage clones were successfully purified with a uniform shape and size. The purified plaque was inoculated into 5 mL of BL21(DE3) culture (~10^9^ CFU/mL) and incubated at 37°C under 200 rpm overnight for phage proliferation. The co-culture mixture was centrifuged at 8,000 rpm for 2 min, and then the supernatant was filtered with 0.22 μm filters to remove bacteria. The phage titer was determined using the double-layer agar method (Han et al., 2022). Briefly, 5 mL of soft LB agar containing 200 μL of overnight BL21(DE3) culture and 100 μL of serial 10-fold diluted phage lysates was plated on a bottom LB agar and incubated at 37°C for 12 h.

### Transmission electron microscopy

The morphology of phage TR1 was observed by transmission electron microscopy (TEM) as described by Han et al. (2022). Shortly, 10 μL of phage concentrate was adsorbed to a formvar film on a carbon-coated copper grid for 15 min, and the adsorbed samples were then negatively stained with 10 μL of 2% phosphotungstic acid for 2 min. Finally, the morphology of phage TR1 was recorded by a JEM-1200EX TEM (Jeol Ltd., Tokyo, Japan) at 80 kV.

### Host range

The host range of phage TR1 was evaluated by the efficiency of the plating (EOP) method against 25 different bacterial strains in our laboratory, including five BL21(DE3) mutants with mutations associated with receptors (Li et al., 2019), *A. baumannii, P. aeruginosa, Salmonella*, and *S. maltophilia*. A total of 5 mL of soft LB agar containing 200 μL of the overnight bacterial culture were plated onto a bottom LB agar plate. Then, 2 μL of serial 10-fold diluted phage TR1 (10^1^-10^8^ PFU/mL) was spotted onto the abovementioned plates. Finally, phage plaque formation was recorded after incubating overnight at 37°C to estimate the sensitivity of different strains to phage TR1. If there were phage plaques on the bacterial lawn, it was considered that phage TR1 had the ability to lyse the bacteria. Conversely, the bacteria were non-sensitive to phage TR1 without phage plaque formation.

### MOI and one-step growth curve

The multiplicity of infection (MOI) represents the ratio of phages to host bacteria during infection. The phage and bacteria were separately subjected to 10-fold serial dilutions. To determine the optimal MOI of phage TR1, the mixture of phage TR1 and host bacteria was prepared with a series of MOI values, including 0.001, 0.01, 0.1, 1, 10, and 100. Taking MOI of 1 as an example, 500 μL of phage solution with a titer of 10^6^ PFU/mL and 500 μL of bacterial culture at a concentration of 10^6^ CFU/mL were thoroughly mixed. Then, the mixture was incubated in 5 mL of LB broth at 37°C with shaking for 5 h. The phage titer was measured at each MOI using the double-layer agar method. The MOI with the highest titer was considered the optimal MOI. Each experiment was performed in triplicate.

The one-step growth curve is fundamental to assessing the infection progress of phages, which reflects the replication cycle of phages in host bacteria. To measure the one-step growth curve, 500 μL of phage TR1 and 500 μL of BL21(DE3) were mixed with the optimal MOI and then incubated at 37°C for 5 min for phage adsorption. Afterward, the incubation was continued at 37°C with shaking, and the samples were taken at 10-min intervals for 180 min. The phage titer was determined by the double-layer agar method. In addition, the burst size and latent period were obtained from the one-step growth curve. The burst size was determined by calculating the ratio between the difference in initial and final phage counts (PFU) during the logarithmic growth phase and the initial quantity of bacteria added (CFU). The experiment was conducted in triplicate.

### Thermal and pH stability

The stability of phages under different temperature and pH conditions is crucial for their storage and practical applications. To assess the thermal stability of phage TR1, 200 μL of the phage suspension (5 × 10^7^ PFU/mL) was placed at various temperatures in the metal bath for 2 h (4°C, 25°C, 37°C, 50°C, 60°C, and 70°C), and then, the phage titer was measured using the double-layer agar method. Similarly, to evaluate the stability of the phages at different pH levels, 200 μL of phage suspension (5 × 10^7^ PFU/mL) was incubated in LB at different pH levels ranging from 1 to 14 for 2 h, which were adjusted with sodium hydroxide and hydrochloric acid. Then, the phage titer was measured using the double-layer agar method as well. Each experiment was performed in triplicate.

### Whole-genome sequencing and bioinformatics analysis

Phage DNA was prepared for sequencing using λ phage Genome DNA Extraction Kit (Leagene Biotechnology Ltd., Beijing, China). Libraries were prepared using NEBNext Ultra™ II DNA Library Prep Kit for Illumina^®^^®^ (New England Biolabs Ltd., Beijing, China) and then sequenced by NovaSeq 6000 (Illumina, San Diego, United States) using NovaSeq 6000 S4 Reagent Kit v1.5 (Illumina, San Diego, United States) with a PE150 sequencing strategy. Trimmomatic (V0.32) program was used to filter the low-quality reads (Bolger et al., 2014). Then, the whole genome of phage TR1 was assembled by SPAdes 3.13.0 (Prjibelski et al., 2020) and subsequently visualized by the software Bandage (Wick et al., 2015). The online tools RAST (https://rast.nmpdr.org/) (Aziz et al., 2008; Overbeek et al., 2014; Brettin et al., 2015) and BLASTp (BLAST: Basic Local Alignment Search Tool) (https://blast.ncbi.nlm.nih.gov/Blast.cgi) were used to predict the open reading frames (ORFs). tRNAscan-SE (http://lowelab.ucsc.edu/tRNAscan-SE/) (Chan et al., 2021) was used to analyze the tRNA sequence. Comparative genomic analysis of phage TR1 sequence was based on the nucleotide database BLASTn. DNA termini and phage packaging mechanisms of TR1 were predicted by Phage Term software (Garneau et al., 2017). A phylogenetic tree of phage TR1 was constructed by an online tool VICTOR (https://ggdc.dsmz.de/victor.php) (Meier-Kolthoff and Goker, 2017) with other 44 phages that have homology to phage TR1 in GenBank (Supplementary Table S1). Genome similarity analysis of phage TR1 among the above 44 phages was conducted by the online tool VIRIDIC (http://rhea.icbm.uni-oldenburg.de/VIRIDIC/) (Moraru et al., 2020). Comparative genomic analysis was performed using Easyfig2.2.3 (Sullivan et al., 2011).

### Construction of recombinant BL21(DE3)

The plasmid containing CRISPR/Cas9 system (Cas9 and interspaced short palindromic repeats) with phage DNA fragments as spacers was transformed into BL21(DE3) to prepare the recombinant cells. In brief, two DNA fragments were designed by the program CRISPOR (http://crispor.tefor.net/) as the spacer from conserved genes of phage TR1, one of which was from ORF62 encoding the major capsid protein (5'-TGGTCTGGCTCTGCACCTCA-3') and the other was from ORF37 encoding the terminase large subunit (5'-GAGGTTTGAATCGATATCTA-3') (Concordet and Haeussler, 2018). Two 20 bp phage DNA fragments were synthesized by Rui Biotech Ltd. (Beijing, China) and separately cloned in plasmid pTCPLS between the two SapI sites. The resulting plasmid containing the spacer from the major capsid protein gene was named PTCPLS-C, and the plasmid containing the other spacer from the terminase large subunit gene was designated as PTCPLS-T. The competent cells of BL21(DE3) were prepared by washing logarithmic phase BL21(DE3) cells three times with 10% (w/v) glycerol. The two plasmids were introduced into competent cells of BL21(DE3) using electroporation, resulting in the generation of two recombinant strains, BL21-C and BL21-T.

### Anti-phage activities of recombinant strains

*EOP assay*. The EOP assay was carried out to determine the anti-phage activities of BL21-C and BL21-T. To conduct this assay, 5 mL of soft LB agar containing 200 μL of the overnight bacterial culture BL21(DE3) was plated onto a bottom LB agar plate. For the soft LB agar with equivalent BL21-C and BL21-T, gentamicin with a final concentration of 20 μg/mL and arabinose with a final concentration of 0.2% were added. Then, 2 μL of serial titers of phages (10^1^-10^8^ PFU/mL) was dropped onto the abovementioned plates containing BL21(DE3), BL21-C, and BL21-T, respectively. Finally, phage plaque formation was recorded after incubating overnight at 37°C to estimate the sensitivity of different strains to phages.

*Growth curves*. The growth curves of BL21-C and BL21-T challenged with phages were investigated to estimate their anti-phage activities under fermentation. In this assay, eight different treatment groups were established, and the treatments are shown in Supplementary Table S2. A and D were negative control groups to monitor the recombinant bacterial cell growth. B and E were positive control groups to observe the effects of phage TR1 infection on recombinant strains. C and F were positive groups to determine the anti-phage activities of recombinant strains under induction. We also investigated phage TR1 infection on BL21(DE3) as references (G) and took BL21(DE3) without phage infection as a blank control group (H). The induction groups were performed with gentamicin at a final concentration of 20 μg/mL and arabinose at a final concentration of 0.2% in LB broth. Phage infection groups were challenged with a titer of 5 × 10^7^ PFU/mL of phage TR1. In addition, phages were added at an MOI value of 1 when the OD_600_ value of the bacterial cultures reached 0.1. The growth of the culture was monitored by measuring the OD_600_ with Multiskan FC (Thermo Fisher Scientific Inc., Shanghai, China) each hour for 8 h with shaking at 37°C. Each experiment was conducted in triplicate.

### Statistical analysis

All data were expressed as mean ± standard deviation and analyzed with a one-way ANOVA test and Tukey's test using GraphPad Prism 8.0.2. A P-value of < 0.05 means that the data were statistically significant. The significance was represented with different symbols, such as ^*^ (P < 0.05), ^**^ (P < 0.01), ^***^ (P < 0.001), and ^****^ (P < 0.0001).

## Results

### Morphology and host range of phage TR1

**Figure 1:**
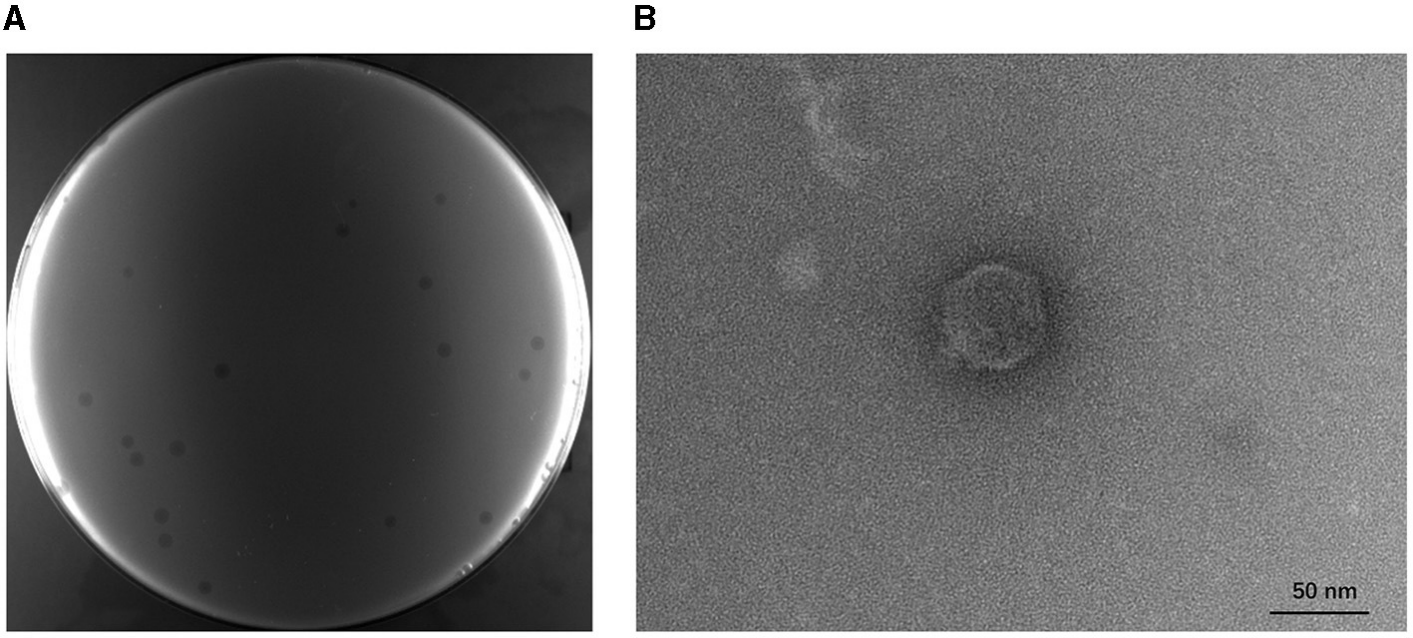
Plaque formation and transmission electron micrograph of phage TR1. **(A)** Plaques formed by phage TR1 on the BL21(DE3) lawn. **(B)** Transmission electron micrograph of phage TR1. Scale bar, 50 nm.

A phage infecting the host strain BL21(DE3) was successfully isolated from the fermentation substrates in the lab of the Chinese Academy of Sciences and was named TR1. It can form transparent plaques with a diameter of 1.24 ± 0.27 mm on the BL21(DE3) lawn, and the plaques were extended by a 0.16 ± 0.02 mm halo (Figure 1A). The TEM image of phage TR1 shows a typical podovirus morphology, which had a capsid with a diameter of ~51.46 ± 2.04 nm and a tail with a length of ~9.31 ± 2.77 nm (Figure 1B).

To examine the lytic activities of phage TR1, a total of 25 bacterial strains, including *E. coli* (17), *A. baumannii* (2), *P. aeruginosa* (2), *Salmonella* (2), and *S. maltophilia* (2), were applied. As shown in Table 1, on the one hand, phage TR1 showed lytic activity against BL21(DE3), including five receptor deletion mutants of BL21(DE3). It suggested that phage TR1 has a negative effect on the fermentation of BL21(DE3) and its derived strains. On the other hand, all other strains were insensitive to phage TR1, indicating its high specificity among different bacterial strains.

### Growth characteristics and stability of phage TR1

**Figure 2:**
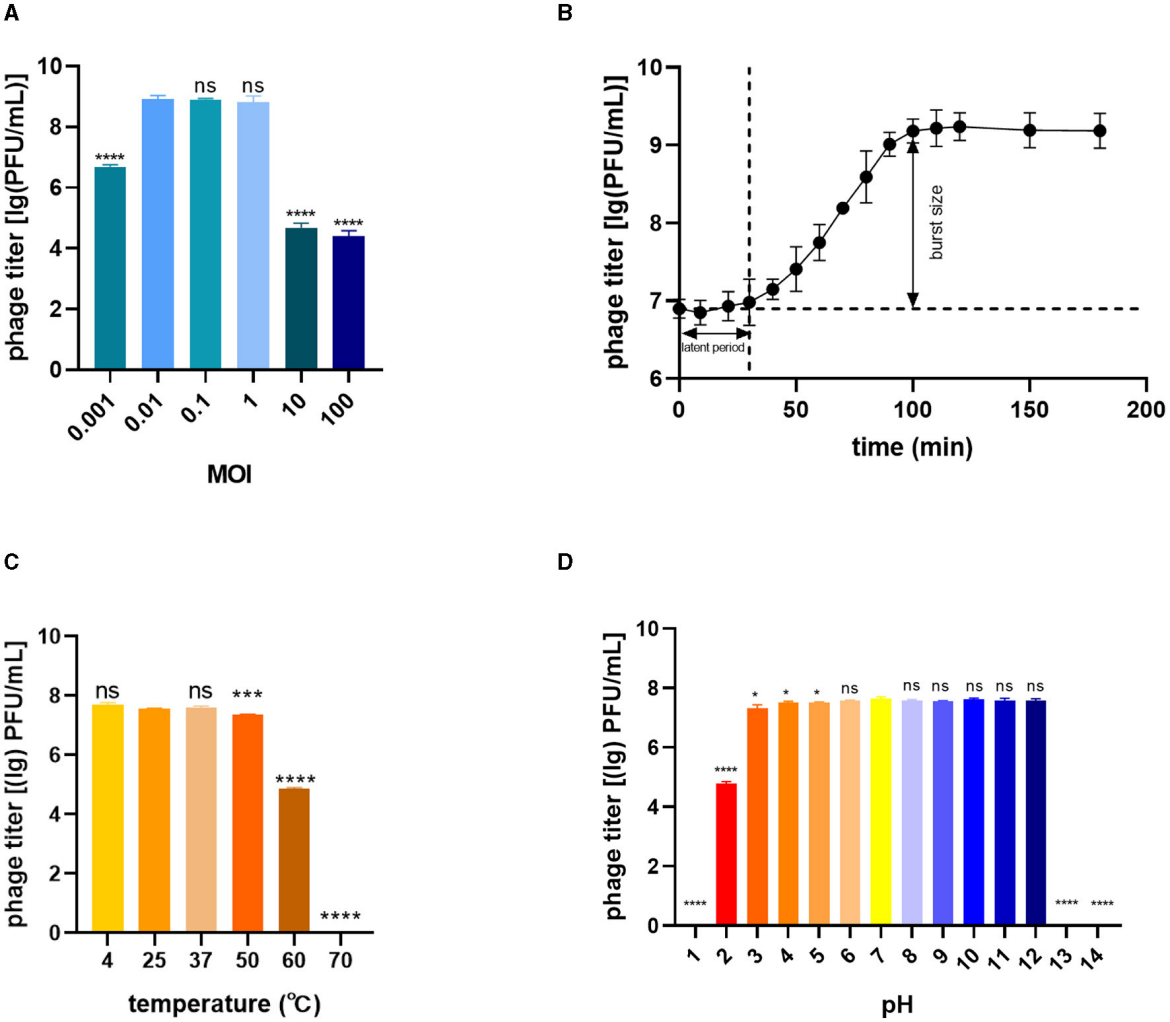
Biological characteristics of phage TR1. **(A)** Phage titers cultured at different MOI values. **(B)** One-step growth curve at a multiplicity of infection (MOI) of 0.1. **(C)** Thermal stability. **(D)** pH sensitivity. Data were expressed as mean ± standard deviation.

To determine the optimal MOI for phage infection, BL21(DE3) was infected with phage TR1 at different MOI values, including 100, 10, 1, 0.1, 0.01, and 0.001. When the MOI was 0.01, 0.1, or 1, the phage titer was significantly higher than other groups, including the MOI values of 0.001, 10, and 100 (Figure 2A), so that the optimal MOI values could be 0.01, 0.1, or 1. Under the optimal MOI of 0.1, the one-step growth curve of phage TR1 was examined for 200 min. As shown in Figure 2B, the latent period of phage TR1 was ~30 min, and the release stage lasted ~70 min. The burst size representing the average number of phage released per bacterium was 151 PFU/cell.

The stability of TR1 was investigated by exposing the phage to different temperature and pH conditions. The titer of phage TR1 had no significant difference when placed at 4°C, 25°C, and 37°C (Figure 2C), indicating its thermal stability between 4°C and 37°C. However, the titer of phage TR1 started to significantly decrease when the temperature reached 50°C or higher, and it was completely inactivated at 70°C, suggesting its thermal instability above 50°C. Similarly, the pH stability of TR1 was measured at different pH levels ranging from 1 to 14. As shown in Figure 2D, the titer of phage TR1 remained at 10^8^ PFU/mL within the pH range of 3 to 12 and sharply decreased exceeding the range, revealing its high tolerance to both strong acidic and alkaline conditions. Phage TR1 was totally inactivated under the pH of 1, 13, and 14.

### Genomic features and phylogenetic analysis

The genomic DNA of phage TR1 is 44,099 bp in length, with 43.8% GC content. The complete annotations of the TR1 genome with supporting evidence are provided in Table 2. After comparison using the online alignment tool BLASTn, phage TR1 had the highest homology to *Enterobacteria* phage vB EcoS IME542 (accession number: NC 048208), with 68% coverage and 99.7% identity, suggesting it to be a novel species. In addition, there were 44 other phages homologous to phage TR1 (Supplementary Table S1), most of which were *Escherichia* phages with only two strains of *Shigella* phages. The software PhageTerm analyzed that phage packaging mechanisms of phage TR1 were headful. The packaging site was at the 3,615bp, located in ORF13, which encodes a hypothetical protein. During the packaging process, the first cut was accomplished at the packaging site in ORF13; however, subsequent cuts were not proceeded until the capsid was full, resulting in a variable position of the termini (Aksyuk and Rossmann, 2011).

**Table 2:** Predicted ORFs in the genome of phage TR1.

**ORF no**.	**Start**	**Stop**	**Strand**	**Predicted function**	**Accession number**	**Length (AA)**	**Best-match BLASTp result**	**Cover**	**Identity**	**E-value**	**Best-match accession number**
1	305	123	-	DUF3667 domain-containing protein	UVD31728.1	60	*Escherichia* phage IMM-001	100%	98.33%	2.00E-23	ATI17072.1
2	833	570	-	Transcriptional repressor	UVD31729.1	87	*Escherichia* phage IMM-001	87%	90.79%	1.00E-44	ATI17068.1
3	1062	826	-	Hypothetical protein	UVD31730.1	78	*Escherichia* phage vB_EcoS_IME542	100%	98.72%	8.00E-37	YP_009824909.1
4	1277	1119	-	Hypothetical protein	UVD31731.1	52	*Escherichia* phage vB_EcoD_SU57	84%	68.18%	6.00E-15	QLF85036.1
5	1428	1270	-	Hypothetical protein	UVD31732.1	52	*Escherichia* phage vB_EcoS_MM01	84%	75.00%	3.00E-15	QBQ80845.1
6	1581	1447	-	Hypothetical protein	UVD31733.1	44	*Escherichia* phage vB_EcoS_MM01	97%	58.14%	1.00E-11	QBQ80840.1
7	1771	1592	-	Hypothetical protein	UVD31734.1	59	*Escherichia* phage AugustePiccard	100%	93.22%	5.00E-34	QXV76203.1
8	1977	1768	-	Methyltransferase	UVD31735.1	69	*Escherichia* phage IMM-001	100%	76.81%	7.00E-31	ATI17060.1
9	2579	2049	-	Hypothetical protein	UVD31736.1	176	*Escherichia* phage vB_EcoD_SU57	99%	69.71%	2.00E-88	QLF85048.1
10	3124	2576	-	Hypothetical protein	UVD31737.1	182	*Escherichia* phage IMM-001	100%	94.51%	2.00E-114	ATI17050.1
11	3744	3595	-	Hypothetical protein	UVD31738.1	49	*Klebsiella* phage vB_KpnD_Opt-79	75%	64.86%	2.00E-07	UGO52827.1
12	3828	4025	-	Hypothetical protein	UVD31739.1	65	*Caudovirales* sp.	72%	74.47%	2.00E-17	DAH81959.1
13	4022	4216	+	Hypothetical protein	UVD31740.1	64	*Escherichia* phage vB_EcoD_SU57	96%	49.21%	1.00E-12	QLF85004.1
14	4218	4421	+	Hypothetical protein	UVD31741.1	67	*Pectobacterium* phage phiTE	98%	39.39%	4.00E-11	YP_007392576.1
15	4421	4582	+	Hypothetical protein	UVD31742.1	53	*Escherichia* phage vB_EcoS-12210I	100%	94.34%	8.00E-31	YP_009900864.1
16	4642	4806	+	Hypothetical protein	UVD31743.1	54	*Escherichia* phage vB_EcoS_MM01	98%	92.45%	1.00E-16	QBQ80900.1
17	4806	5159	+	Hypothetical protein	UVD31744.1	117	*Escherichia* phage JeanPiccard	99%	87.07%	2.00E-71	QXV80820.1
18	5233	6816	+	DUF3987 domain-containing protein	UVD31745.1	527	*Escherichia* phage vB_EcoS_IME542	100%	96.96%	0.00E+00	YP_009824889.1
19	6820	7155	+	Hypothetical protein	UVD31746.1	111	*Escherichia* phage vB_EcoS_IME542	100%	91.89%	3.00E-70	YP_009824888.1
20	7553	7167	+	u-spanin	UVD31747.1	128	*Escherichia* phage JeanPiccard	93%	71.54%	5.00E-49	QXV80817.1
21	8035	7550	-	Lysozyme	UVD31748.1	161	*Escherichia* phage vB_EcoS_IME542	100%	99.38%	6.00E-88	YP_009824886.1
22	8328	8035	-	Holin	UVD31749.1	97	*Escherichia* phage vB_EcoS_IME542	100%	100.00%	2.00E-48	YP_009824885.1
23	8552	8379	-	Hypothetical protein	UVD31750.1	57	*Escherichia* phage vB_EcoS_FP	100%	84.48%	1.00E-27	QLF80606.1
24	9165	8611	-	ATPase	UVD31751.1	184	*Escherichia* phage vB_EcoD_SU57	99%	76.50%	2.00E-101	QLF84977.1
25	9763	9215	-	Polynucleotide kinase	UVD31752.1	182	*Escherichia* phage P818	98%	72.63%	7.00E-92	UOX38508.1
26	10974	9841	-	DNA binding protein	UVD31753.1	377	*Caudovirales* sp.	100%	86.21%	0.00E+00	DAF69443.1
27	11307	11056	-	Hypothetical protein	UVD31754.1	83	*Escherichia* phage vB_EcoD_SU57	98%	92.68%	3.00E-51	QLF85014.1
28	11552	11310	-	Hypothetical protein	UVD31755.1	80	*Escherichia* phage vB_EcoS_ACG-M12	98%	84.81%	1.00E-44	YP_006987877.1
29	11674	11549	-	Hypothetical protein	UVD31756.1	41	*Escherichia* phage vB_EcoD_SU57	100%	85.37%	8.00E-18	QLF85015.1
30	11895	11674	-	Hypothetical protein	UVD31757.1	73	*Escherichia* phage vB_EcoS_Rogue1	94%	88.41%	4.00E-38	YP_007112254.1
31	12095	11895	-	Hypothetical protein	UVD31758.1	66	*Escherichia* phage vB_EcoS_FP	100%	87.88%	5.00E-34	QLF80597.1
32	12593	12174	-	Nuclease	UVD31759.1	139	*Caudovirales* sp.	100%	92.81%	3.00E-93	DAH81930.1
33	14584	12590	-	DNA helicase	UVD31760.1	664	*Escherichia* phage AugustePiccard	100%	92.47%	0.00E+00	QXV76173.1
34	14681	15154	-	Transcription regulator	UVD31761.1	157	*Escherichia* phage vB_EcoS_ESCO41	100%	87.90%	2.00E-97	YP_009789963.1
35	15207	15686	+	HNH endonuclease	UVD31762.1	159	*Escherichia* phage vB_EcoS_IME542	100%	100.00%	5.00E-115	YP_009824873.1
36	15683	16606	+	DNA primase	UVD31763.1	307	*Escherichia* phage vB_EcoS_IME542	100%	100.00%	0.00E+00	YP_009824872.1
37	16694	19171	+	Tail fiber protein	UVD31764.1	825	*Escherichia* phage CJ19	26%	81.02%	7.00E-89	QIW88869.1
38	19636	19208	+	Single-stranded DNA binding protein	UVD31765.1	142	*Caudovirales* sp.	76%	82.41%	3.00E-62	DAF69386.1
39	20007	19633	-	HNH endonuclease	UVD31766.1	124	*Escherichia* phage vB_EcoD_Opt-719	85%	40.57%	1.00E-15	UGO52666.1
40	20837	20187	-	Recombinase	UVD31767.1	216	*Escherichia* phage IMM-001	100%	91.67%	7.00E-147	ATI17130.1
41	21385	20894	-	HNH endonuclease	UVD31768.1	163	*Escherichia* phage vB_EcoS_IME542	100%	99.39%	1.00E-115	YP_009824939.1
42	22343	21372	-	Exodeoxyribonuclease 8	UVD31769.1	323	*Caudovirales* sp.	99%	81.56%	0.00E+00	DAH81927.1
43	22694	22347	-	Hypothetical protein	UVD31770.1	115	*Escherichia* phage vB_EcoS_IME542	100%	98.26%	2.00E-78	YP_009824937.1
44	23087	23317	-	Lipoprotein	UVD31771.1	76	*Escherichia* phage vB_EcoS_CEB_ EC3a	100%	73.75%	3.00E-35	YP_009789334.1
45	23317	24279	+	DUF6453 family protein	UVD31772.1	320	*Escherichia* phage vB_EcoS_IME542	100%	97.81%	0.00E+00	YP_009824935.1
46	27695	24306	+	Tail fiber protein	UVD31773.1	1129	*Escherichia* phage vB_EcoD_SU57	99%	93.62%	0.00E+00	QLF84996.1
47	28348	27776	-	Tail assembly protein	UVD31774.1	190	*Escherichia* phage vB_EcoS_IME542	100%	100.00%	9.00E-137	YP_009824933.1
48	29087	28329	-	C40 family peptidase	UVD31775.1	252	*Escherichia* phage vB_EcoS_IME542	100%	100.00%	0.00E+00	YP_009824932.1
49	29856	29101	-	Minor tail protein L	UVD31776.1	251	*Escherichia* phage vB_EcoS_IME542	100%	99.60%	0.00E+00	YP_009824931.1
50	30246	29896	-	Minor tail protein	UVD31777.1	116	*Escherichia* phage vB_EcoD_SU57	100%	91.38%	3.00E-75	QLF85001.1
51	30718	30248	-	HNH endonuclease	UVD31778.1	156	*Escherichia* phage vB_EcoS_IME542	100%	99.36%	6.00E-98	YP_009824929.1
52	33558	30790	-	Tail type measure protein	UVD31779.1	922	*Escherichia* phage AugustePiccard	100%	92.08%	0.00E+00	QXV76152.1
53	33847	33596	-	TfmS	UVD31780.1	83	*Escherichia* phage IMM-001	100%	93.98%	2.00E-52	ATI17106.1
54	34224	33910	-	Tail assembly chaperone	UVD31781.1	104	*Escherichia* phage IMM-001	100%	99.04%	3.00E-69	ATI17105.1
55	34650	34270	-	Hypothetical protein	UVD31782.1	126	*Escherichia* phage AugustePiccard	96%	31.45%	4.00E-03	QXV76149.1
56	35421	34762	-	Tail tube protein	UVD31783.1	219	*Caudovirales* sp.	99%	92.66%	5.00E-138	DAF69375.1
57	35838	35437	-	Tail completion protein	UVD31784.1	133	*Caudovirales* sp.	100%	96.99%	7.00E-92	DAH81925.1
58	36265	35828	-	Putative tail component	UVD31785.1	145	*Caudovirales* sp.	100%	91.72%	1.00E-92	DAH81924.1
59	36629	36258	-	Head tail attachment	UVD31786.1	123	*Caudovirales* sp.	100%	95.93%	6.00E-80	DAF69372.1
60	37027	36626	-	Head to tail adaptor	UVD31787.1	133	*Caudovirales* sp.	100%	90.23%	3.00E-82	DAH81918.1
61	37314	37069	-	Hypothetical protein	UVD31788.1	81	*Escherichia* phage vB_EcoS-IME253	100%	100.00%	3.00E-51	YP_009789207.1
62	38351	37407	-	Major capsid protein	UVD31789.1	314	*Escherichia* phage AugustePiccard	100%	93.63%	0.00E+00	QXV76142.1
63	38988	38494	-	Scaffolding protein SbcC like	UVD31790.1	164	*Caudovirales* sp.	95%	73.49%	7.00E-75	DAH81923.1
64	40112	39000	-	Prohead serine protease	UVD31791.1	370	*Caudovirales* sp.	99%	70.46%	1.00E-151	DAH81980.1
65	41376	40102	-	Portal protein	UVD31792.1	424	*Escherichia* phage vB_EcoS_FP	99%	76.78%	0.00E+00	QLF80552.1
66	42868	41426	-	Terminase large subunit	UVD31793.1	480	*Escherichia* phage vB_EcoS_FP	100%	94.17%	0.00E+00	QLF80551.1
67	43506	43000	-	HNH endonuclease	UVD31794.1	168	*Salmonella* phage Segz_1	94%	46.84%	3.00E-37	YP_010053381.1
68	44099	43575	-	Terminase small subunit	UVD31795.1	174	*Escherichia* phage IMM-001	100%	93.10%	8.00E-119	ATI17076.1
tRNA	397	325	-	tRNA			not hits				

**Figure 3:**
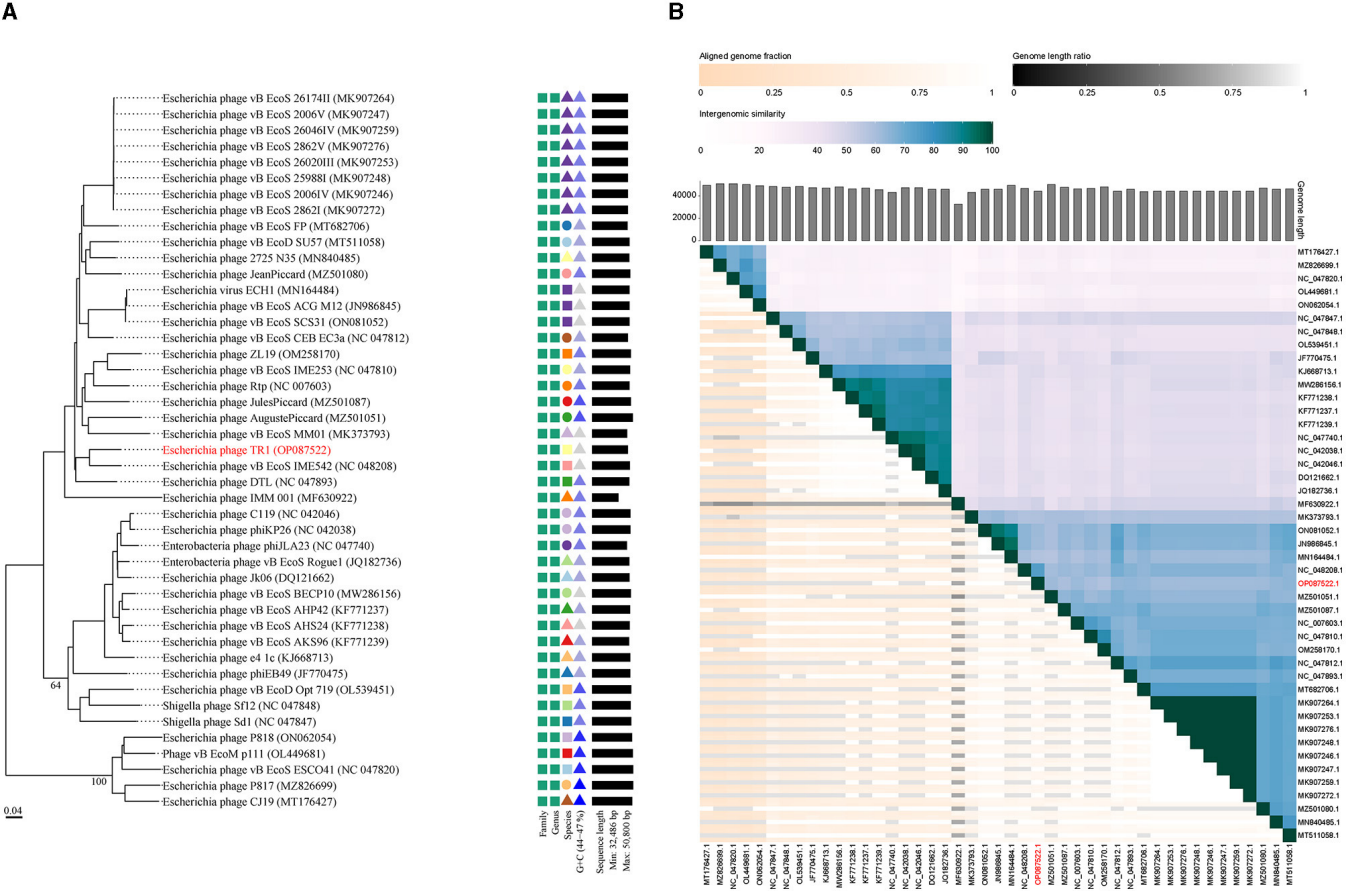
Phylogenetic analysis and ANI of phage TR1. **(A)** Phylogenetic tree was conducted by an online tool VICTOR using the whole sequence, showing the evolutionary relationship between phage TR1 and other phages. **(B)** Percentage similarity of sequence among phages calculated using VIDIRIC. The horizontal and vertical coordinates indicate phages and their corresponding GenBank accession number, and phage TR1 was highlighted.

A phylogenetic tree was constructed using the complete genome sequences with a total of 45 phages in GenBank that had homology with phage TR1 to study the relative distance among them. Phage TR1 was on the same clade with *Enterobacteria* phage vB_EcoS_IME542, whose host was Rosetta (DE3) (Figure 3A). According to the classification of VICTOR, all of them belonged to the family *Drexlerviridae*, subfamily *Braunvirinae*, and genus *Christensenvirus*, while phage TR1 was an independent species (Figure 3A). In addition, the similarity analysis showed that phage TR1 had a maximum similarity of 74.4% with *Enterobacteria* phage vB_EcoS_IME542 (accession number: NC 048208) (Figure 3B), representing it was a novel phage.

### Functional ORFs analysis

**Figure 4:**
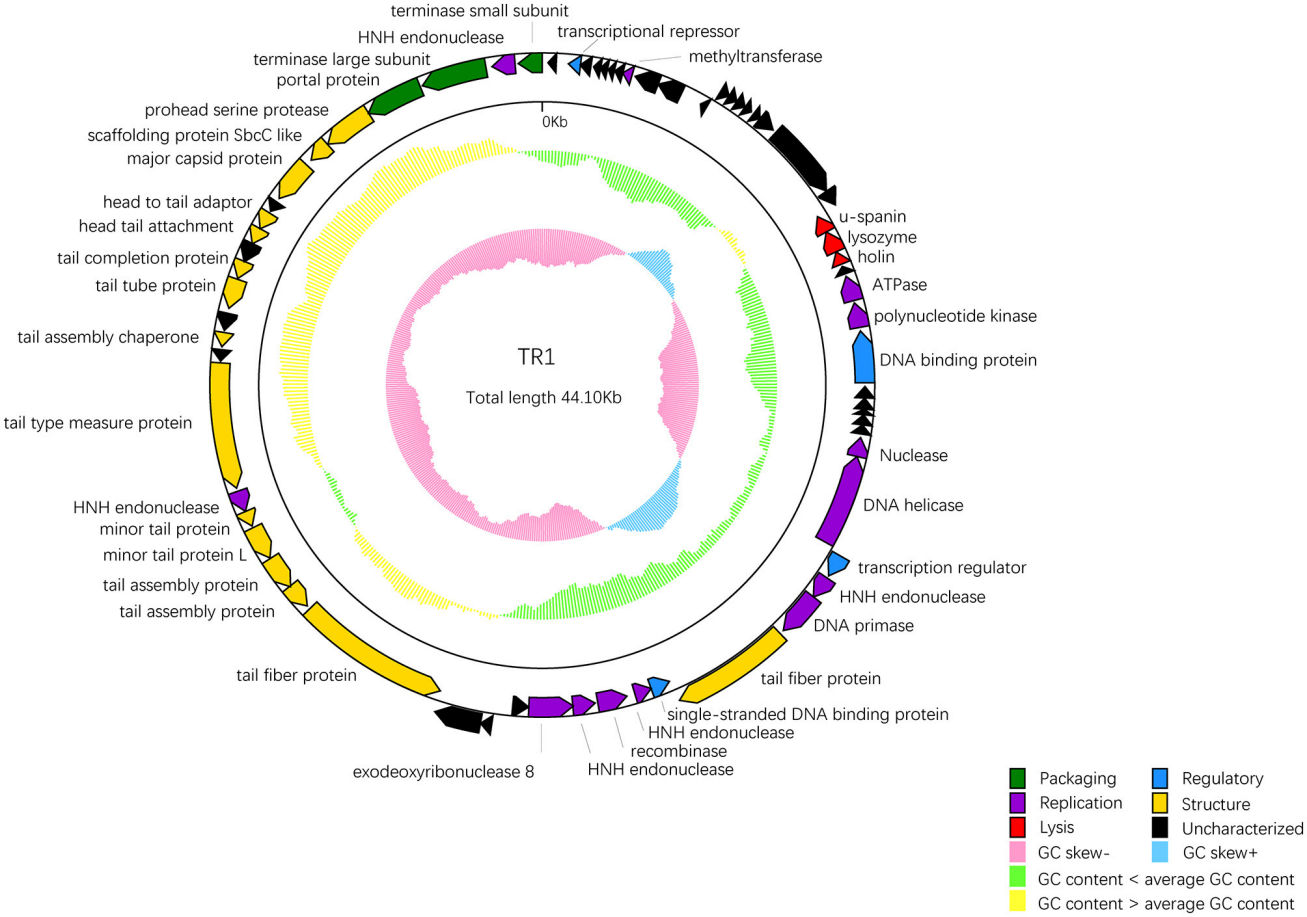
The whole genetic map of phage BUCT609. Colors distinguish different functional genes, and arrows represent ORF directions. A total of 68 ORFs of phage TR1 were divided into 6 modules: packaging (green), regulatory (blue), replication (purple), structure (yellow), lysis (red), and uncharacterized (black). The middle circle represents GC content [outward for larger than the whole-genome average GC content (yellow) and inward for the opposite (green)]. The innermost circle represents the G + C tilt of G–C/G + C [outward for >0 (blue) and inward for <0 (pink)].

Among 68 ORFs, 44 ORFs are similar to genes encoding functional proteins with known functions, while the remaining 24 ORFs have some homology to genes encoding hypothetical proteins whose functions are unknown (Table 2). In addition to the 68 ORFs mentioned above, RAST annotation and tRNA scan-SE analysis revealed the presence of a 67 bp arginine tRNA, which could compensate for the translation deficiency of the host tRNA. All 44 ORFs, homologous to genes with known functions, could be divided into five functional modules: packaging, regulatory, replication, structure, and lysis (Figure 4).

Package-relative genes are ORF66 and ORF68, which respectively encodes the large and small subunits of terminase. The terminase of phages is a protein involved in the transposition of DNA within the capsid protein. It is mainly composed of an oligomer consisting of a large subunit and a small subunit and plays an important role in genome cleavage and transfer activity (Ray et al., 2009). In addition, there are two DNA-binding proteins that may recruit other regulatory factors to form complexes by specifically binding with DNA, thereby regulating gene expression and controlling cell growth, differentiation, and function (Hernandez and Richardson, 2019). The single-stranded DNA binding protein may also bind and protect single-stranded DNA molecules from degradation and damage (Hernandez et al., 2022). ORF33 encodes a DNA helicase protein, whose main function is to unwind the double helix structure of DNA, making it easier to replicate while the protein encoded by ORF36 has DNA primase activities and is involved in DNA replication. Markedly, five endonuclease ORFs (ORF35, 39, 41, 51, and 67) exist in the phage TR1 genome. The enzyme initiates transfer of DNA elements and acts as a maturase (Stoddard, 2005). The protein encoded by ORF24 has ATPase activity that has an effect on the process of DNA repair and recombination (Nandi and Whitby, 2012).

As for the structural proteins, ORF62 encodes a capsid protein that wraps around the genetic material DNA of the phage (Prevelige Jr and Cortines, 2018). Apart from ORF62, most structural proteins are associated with tail structures including tail fiber protein, tail type measure protein, tail assembly chaperone protein, tail tube protein, and tail completion protein. Tail fiber proteins usually participate in the recognition, adhesion, and penetration of the host bacteria. It also determines the host range of phages (Taslem Mourosi et al., 2022). The tail type measure protein could measure the length of the phage tail to ensure the consistency of tail length. Consistency in tail length is critical for the successful infection of the phage as tail length affects the interaction of the phage with host cells and the efficiency of phage injection (Cumby et al., 2015). In addition, tail assembly chaperone protein helps to maintain the correct structure during the assembly process of the phage tail. It can interact with other tail proteins to prevent them from improperly combining during the assembly process, thereby ensuring normal replication of the phage (Xu et al., 2014). The tail completion protein is encoded by ORF57 that involves in the final stage of the tail assembly, promoting the proper folding and assembly of tail components to ensure the normal formation of the tail (Zhao et al., 2003).

ORF20-22 are lysis-related genes, encoding u-spanin, lysozyme, and holin protein, respectively. The u-spanin (ORF20) is one of the topological structures of the spanin that means unimolecular, and it is crucial for disrupting the outer membrane to promote infection (Kongari et al., 2018). Holin proteins can enable the release of endolysins to cross the inner membrane (Cahill and Young, 2019). Lysozyme provokes cell lysis and facilitates the release of phages by breaking the bacterial cell wall (Fastrez, 1996).

### Bacteria with phage resistance

**Figure 5:**
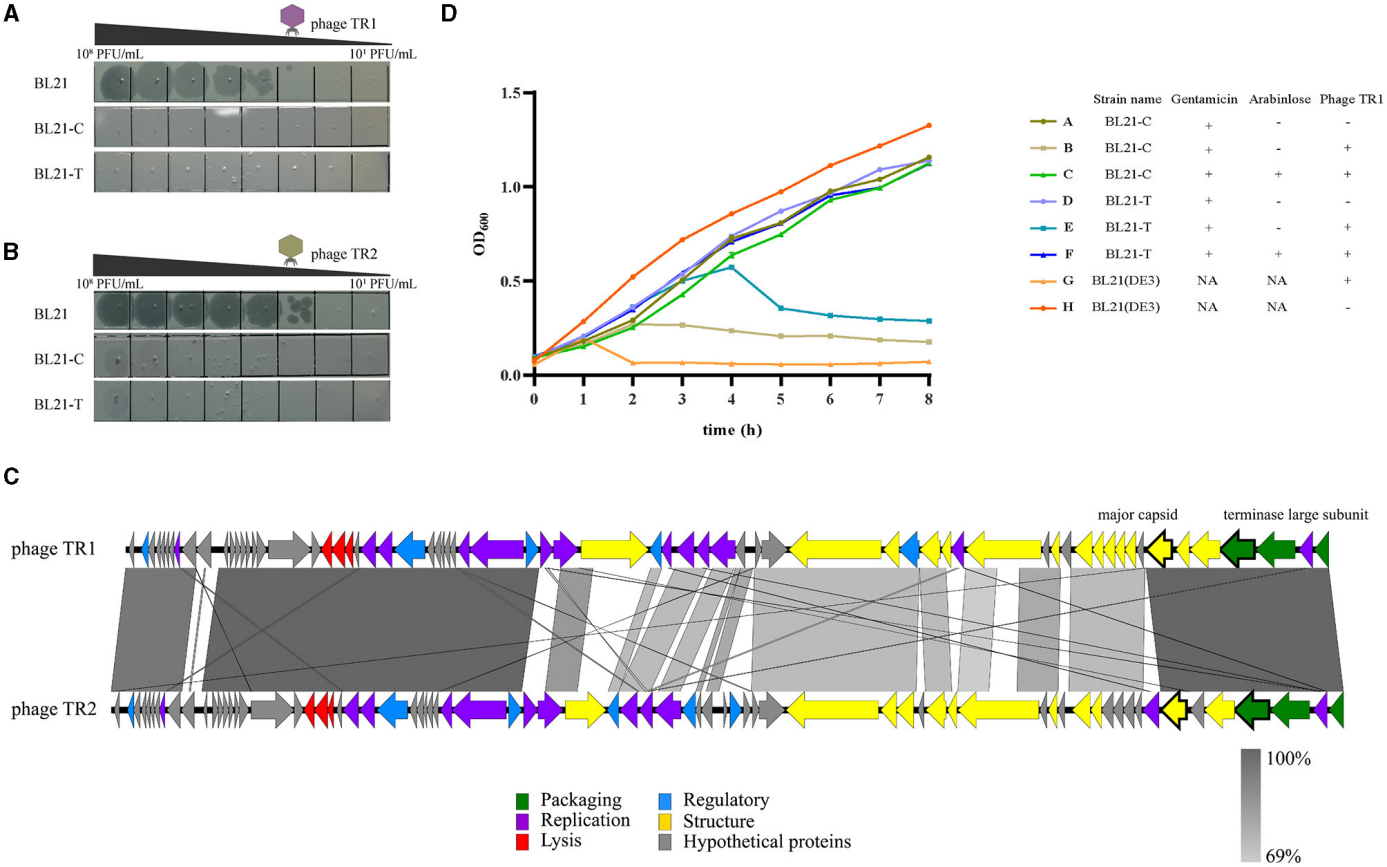
Anti-phage activities of recombinant BL21(DE3) strains. **(A)** The plaque formation of phage TR1 in strains BL21(DE3), BL21-C, and BL21-T. **(B)** The plaque formation of phage TR2 in strains BL21(DE3), BL21-C, and BL21-T. **(C)** Genome-wide sequence comparison between phage TR1 and phage TR2. **(D)** The growth curves of BL21(DE3), BL21-C, and BL21-T with different treatments. In total, 0.2% arabinose was added to induce the activity of the Cas9 protein. Regardless of whether the activity of Cas9 protein was induced, gentamicin was added at a final concentration of 20 μg/mL as a selective agent for the plasmid. Phage infection groups were challenged with a titer of 5 × 10^7^ PFU/mL of phage TR1 at a MOI of 1. Data were expressed as mean ± standard deviation.

Based on the genome sequence of phage TR1, two spacers were designed and inserted into plasmid pTCPLS containing the CRISPR/Cas9 system. In order to validate the feasibility of the CRISPR/Cas9 system-based strategy to eliminate phage contamination, both anti-phage activities and growth stability of BL21(DE3) harboring the recombinant plasmid were tested. First, an EOP experiment was conducted to determine the phage-resistant performance of the recombinant bacterial strains. Both recombinant strains BL21-C and BL21-T, with specific spacer targeting the major capsid gene and the terminase large subunit gene of phage TR1, conferred 10^6^-fold protection against phage TR1 (Figure 5A). Subsequently, growth curves of BL21(DE3) and its recombinant strains with different treatments were monitored for 8 h at an MOI of 1. Without phage TR1 infection, growth curves of the recombinant strains showed a similar growing pattern to that of BL21(DE3), suggesting that the introduction of plasmid pLPCTS had no effects on bacterial growth stability. Conversely, OD_600_ of BL21(DE3) rapidly decreased 1 h after phage TR1 infection, and the bacteria remained inactive for 8 h. As for recombinant strains, both BL21-C and BL21-T could struggle to survive for 2-4 h despite no induction but eventually failed to fight against phage infection with reduced OD_600_ values. On the other hand, BL21-C and BL21-T performed strong immunity to phage TR1 after induction, and their growth curves were nearly consistent with that of the original strain BL21(DE3) (Figure 5D).

To examine the application of the recombinant strains to combat other phages, we employed BL21(DE3) as the host and isolated another novel phage, named TR2, from the fermentation substrates in China Pharmaceutical University Nanjing First Hospital (118°47′3.68″E, 32°01′1.87″N). After a genome-wide sequence comparison between these two phages, phage TR2 (GenBank accession number: OP251154) shared 75% overall genome similarity with TR1 (Figure 5C). Even though TR1 and TR2 phages share only 75% overall genome similarity, the ORFs in which the spacers target (i.e., the major capsid protein and the terminase large subunit) are extremely similar with an identity of 96.51% and 99.79%, respectively. After EOP analysis, phage TR2 was unable to form plaques on either BL21-C or BL21-T bacterial lawn (Figure 5B), indicating that both recombinant strains showed equally strong anti-phage activities toward phage TR2 infection. Therefore, we can construct broad-spectrum anti-phage recombinant strains based on a highly conversed sequence, which can be inserted into the CRISPR/Cas9 system as spacers to target extensive phages containing this DNA fragment.

## Discussion

**Figure 6:**
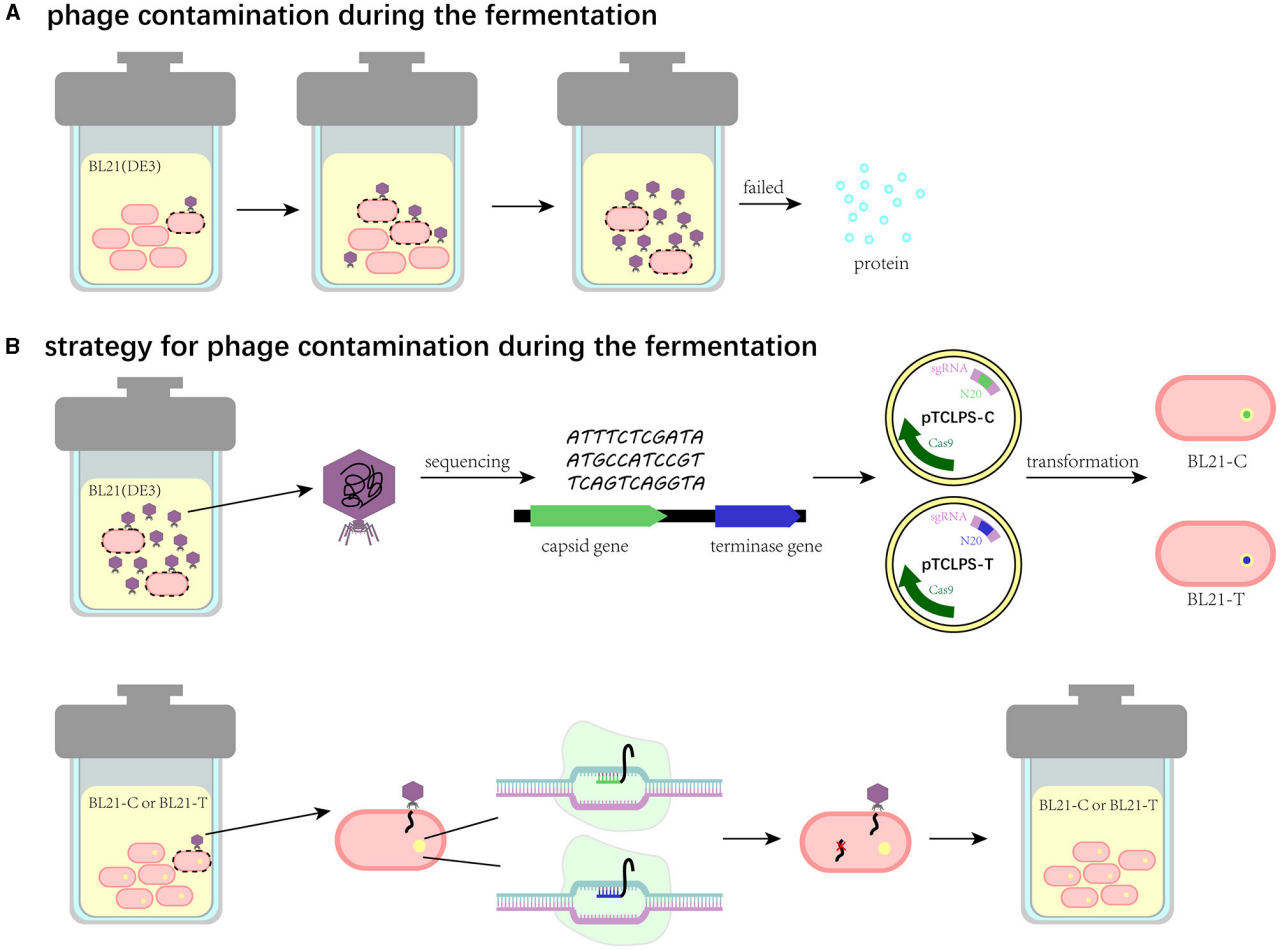
Schematic illustration of the CRISPR/Cas9 system-based strategy to eliminate phage contamination in the fermentation system. **(A)** Phage contamination and its effects during the fermentation. **(B)** The CRISPR/Cas9 system-based strategy to eliminate phage contamination in the fermentation system.

Phage contamination represents one of the greatest challenges in bacterial growth, especially in industrial fermentation, which can lead to fermentation failure and enormous financial losses (Figure 6A). Thus, these effects have accentuated the significance to prevent phage proliferation during the fermentation process. Notably, bacteria have a wide variety of strategies to prevent phage proliferation by themselves. First, bacteria can alter receptors to prevent phage adsorption. Correspondingly, the receptor-binding proteins of phages are mutated with a high frequency (Hampton et al., 2020). Additionally, RM systems can employ DNA methylation signals to discriminate self genome to foreign DNA. Specifically, it can use methyltransferases to methylate self-DNA and adopt restriction endonucleases to cleave the unmethylated DNA. Meanwhile, phages can exploit modified bases such as acetamidation, hydroxymethylation, and glycosylation to escape from the restriction of endonuclease (Loenen et al., 2014). In contrast to adsorption inhibition and RM, Abi anti-phage systems protect bacterial populations by triggering self-destruction mechanisms to disrupt the growth of phages (van Houte et al., 2016). CRISPR/Cas system, as one of the most widely prevalent natural immune mechanisms in bacteria, can generate memories, which capture a short sequence (20–60 bp) of phages and store it in spacers (Barrangou et al., 2007). Once bacteria are infected with phages, they can stimulate the nuclease activity of Cas proteins to undermine the integrity of invading phage genome effectively and specifically by recognizing (Garneau et al., 2010). Phage-resistant strains based on defense strategies are attracting immense attention as alternative approaches to decontaminate phages in the biotechnology and food industries. Zhou et al. reported a strategy by utilizing one of the RM systems called SspBCDE to construct phage-resistant *E. coli* strains and performed great protection against extensive phages (Zou et al., 2022). Similarly, we propose a CRISPR/Cas9 system-based strategy to block phage entry within manufacturing facilities (Figure 6B). In brief, the contaminated phage in fermentation substrates can be isolated, characterized, and sequenced to obtain its biological characteristics and genome information. Then, the conserved genes, such as the capsid gene or terminase gene, can be used to design spacers, and the recombinant plasmid harboring the CRISPR/Cas9 system is transformed into the bacterial cell for fermentation. This strategy is expected to enable recombinant bacterial cells to target and degrade phage DNA after induction so that phage DNA replication can be inhibited and thus the contaminated phage is unable to produce offspring. To examine the feasibility and application of this strategy, we isolated the contaminated phage TR1 from the fermentation substrates, which can be classified as a new species of the family *Drexlerviridae*, subfamily *Braunvirinae*, and genus *Christensenvirus*. The burst size of this phage was 151 PFU/cell, much more than that of phage vB_EcoS_SCS31 (117 PFU/cell) and phage vB_KpnS_MK54 (60 PFU/cell) (Alexyuk et al., 2022; Lu et al., 2022), suggesting its strong fecundity in the fermentation system. Host range assay indicated its high specificity to industrial BL21(DE3) and its receptor-related mutants (Li et al., 2019). Therefore, we took phage TR1 as an example to develop its engineered phage-resistant *E. coli* host. After obtaining the genome sequence of phage TR1, we designed and constructed recombinant plasmids pTCPLS-C and pTCPLS-T based on the most conserved genes encoding major capsid (protein id: UVD31789.1) and terminase large subunit (protein id: UVD31793.1). After induction, both EOP and growth curves of recombinant bacterial strains revealed their strong anti-phage abilities, suggesting that our CRISPR/Cas9 system-based strategy worked. In addition, the phage immunity of these two recombinant strains was also observed when attacked by another contaminated phage TR2.

The strategy was once regarded to have limited effects on a narrow range of phages since the specific target sequences are not present in all phage DNA (Zou et al., 2022). However, the ongoing arms race between bacteria and phages (Shaer Tamar and Kishony, 2022) makes a “one-and-done” strategy impossible, and our strategy is resilient to counteract ever-changing phages in various fermentation systems. First, although the target sequences are not present in all phage DNA, the nanopore genome sequencing technique (Gorzynski et al., 2022) can be adopted to obtain the genome sequence of contaminated phages within 8 h. Then, personalized CRISPR/Cas9 system-based recombinant strains for the fermentation factory could be constructed to fight against one or more contaminated phages timely (Han et al., 2013). In addition, some counterstrategies should be considered in the design of recombinant strains, which can efficiently respond to the ongoing arms race between bacteria and phages. For example, since the plasmid-encoded defense system can confer bacterial cells' immunity to phages easily and conveniently, the ingenious design of multiple spacers in cascades or rotational introduction of CRISPR/Cas system in the recombinant plasmid can prevent phages from invading this defense system. Furthermore, due to the widespread presence of the CRISPR/Cas system in diverse bacterial strains, the scheme of constructing engineered strains can be extended to diverse strains for fermentation use, such as *Bacillus subtilis* (Jakutyte-Giraitiene and Gasiunas, 2016). However, the strategy may be limited sometimes since plasmids are unstable within bacterial cells and impose a burden on bacteria with additional metabolic costs (Summers et al., 1993; Santos et al., 2013). To generate stable phage-resistant strains, Zhou et al. proposed a strategy by integrating the defense system into bacterial chromosomes (Zou et al., 2022), which may be a more effective and sustainable strategy in the future to fight against phage contamination in industrial fermentation. Apart from CRISPR/Cas system, some other phage defense mechanisms can be introduced in anti-phage strategies, such as Abi (Durmaz and Klaenhammer, 1995), antisense mRNA (Walker and Klaenhammer, 2000), and RM systems (Durmaz and Klaenhammer, 1995; Gabs and Josephsen, 2003), and numerous newly discovered mechanisms also possess the potential to be exploited.

## Conclusion

In conclusion, we isolated a novel phage TR1 from a fermentation system, which specifically infected industrial *E. coli* BL21(DE3) and its mutants. This phage had a strong fecundity in the fermentation system due to its large burst size of 151 PFU/cell. Accordingly, we constructed phage-resistant *E. coli* strains by the introduction of the CRISPR/Cas9 defense system to fight against phage TR1. Under exposure to phages, both recombinant strains BL21-C and BL21-T performed 10^6^-fold protection in the EOP assay and showed a similar growing pattern to that of BL21(DE3) without phage pressure. Far more than desirable phage resistance, our strategy is resilient to respond to ever-changing phages in industrial fermentation environments. On the one hand, we can rapidly obtain the genome sequence of the contaminated phage by leveraging the immediacy of nanopore genome sequencing and correspondingly design a personalized CRISPR/Cas system to control it within 2 days. On the other hand, some counterstrategies, such as the ingenious design of multiple spacers in cascades in the recombinant CRISPR/Cas system-containing plasmid, can be adopted to combat the ongoing arms race between bacteria and phages. More importantly, our research opens up new perspectives to employ phage defense elements to develop extensive phage-resistant industrial strains in diverse biotechnological applications.

## Data availability statement

The datasets presented in this study can be found in online repositories. The names of the repository/repositories and accession number(s) can be found in the article/Supplementary material.

## Author contributions

YD: data curation, writing—original draft, investigation, and validation. YH: data curation. HF: supervision and writing—reviewing and editing. LS, XA, and SX: supervision, validation and writing—reviewing and editing. ML: conceptualization, methodology, supervision, validation, and writing—reviewing and editing. YT: conceptualization, supervision, validation, and writing—reviewing and editing. All authors contributed to the article and approved the submitted version.

## References

[B1] Aksyuk A. A. Rossmann M. G. (2011). Bacteriophage assembly. Viruses 3, 172–203. 10.3390/v3030172 21994726 PMC3185693

[B2] Alexyuk P. Bogoyavlenskiy A. Alexyuk M. Akanova K. Moldakhanov Y. Berezin V.. (2022). Isolation and characterization of lytic bacteriophages active against clinical strains of *E. coli* and development of a phage antimicrobial cocktail. Viruses 14, 2381. 10.3390/v14112381 36366479 PMC9697832

[B3] Azam A. H. Tanji Y. (2019). Bacteriophage-host arm race: an update on the mechanism of phage resistance in bacteria and revenge of the phage with the perspective for phage therapy. Appl. Microbiol. Biotechnol. 103, 2121–2131. 10.1007/s00253-019-09629-x 30680434

[B4] Aziz R. K. Bartels D. Best A. A. DeJongh M. Disz T. Edwards R. A.. (2008). The RAST Server: rapid annotations using subsystems technology. BMC Genomics 9, 75. 10.1186/1471-2164-9-75 18261238 PMC2265698

[B5] Baltz R. H. (2018). Bacteriophage-resistant industrial fermentation strains: from the cradle to CRISPR/Cas9. J. Ind. Microbiol. Biotechnol. 45, 1003–1006. 10.1007/s10295-018-2079-4 30191429

[B6] Barrangou R. Fremaux C. Deveau H. Richards M. Boyaval P. Moineau S.. (2007). CRISPR provides acquired resistance against viruses in prokaryotes. Science 315, 1709–1712. 10.1126/science.1138140 17379808

[B7] Bertozzi Silva J. Storms Z. Sauvageau D. (2016). Host receptors for bacteriophage adsorption. FEMS Microbiol. Lett. 363, fnw002 10.1093/femsle/fnw002 26755501

[B8] Bolger A. M. Lohse M. Usadel B. (2014). Trimmomatic: a flexible trimmer for illumina sequence data. Bioinformatics 30, 2114–2120. 10.1093/bioinformatics/btu170 24695404 PMC4103590

[B9] Bondy-Denomy J. Qian J. Westra E. R. Buckling A. Guttman D. S. Davidson A. R.. (2016). Prophages mediate defense against phage infection through diverse mechanisms. ISME J. 10, 2854–2866. 10.1038/ismej.2016.79 27258950 PMC5148200

[B10] Brettin T. Davis J. J. Disz T. Edwards R. A. Gerdes S. Olsen G. J.. (2015). RASTtk: a modular and extensible implementation of the RAST algorithm for building custom annotation pipelines and annotating batches of genomes. Sci. Rep. 5, 8365. 10.1038/srep08365 25666585 PMC4322359

[B11] Cahill J. Young R. (2019). Phage lysis: multiple genes for multiple barriers. Adv. Virus Res. 103, 33–70. 10.1016/bs.aivir.2018.09.003 30635077 PMC6733033

[B12] Chan P. P. Lin B. Y. Mak A. J. Lowe T. M. (2021). tRNAscan-SE 2.0: improved detection and functional classification of transfer RNA genes. Nucleic Acids Res. 49, 9077–9096. 10.1093/nar/gkab688 34417604 PMC8450103

[B13] Concordet J. P. Haeussler M. (2018). CRISPOR: intuitive guide selection for CRISPR/Cas9 genome editing experiments and screens. Nucleic Acids Res. 46, W242–W245. 10.1093/nar/gky354 29762716 PMC6030908

[B14] Cumby N. Reimer K. Mengin-Lecreulx D. Davidson A. R. Maxwell K. L. (2015). The phage tail tape measure protein, an inner membrane protein and a periplasmic chaperone play connected roles in the genome injection process of E. coli phage HK97. Mol. Microbiol. 96, 437–447. 10.1111/mmi.12918 25532427

[B15] Durmaz E. Klaenhammer T. R. (1995). A starter culture rotation strategy incorporating paired restriction/ modification and abortive infection bacteriophage defenses in a single *Lactococcus lactis* Strain. Appl. Environ. Microbiol. 61, 1266–1273. 10.1128/aem.61.4.1266-1273.1995 16534987 PMC1388405

[B16] Fastrez J. (1996). Phage lysozymes. EXS 75, 35–64. 10.1007/978-3-0348-9225-4_3 8765293

[B17] Gabs S. Josephsen J. (2003). Improvement of phage defence in *Lactococcus lactis* by introduction of the plasmid encoded restriction and modification system LlaAI. Lett. Appl. Microbiol. 36, 332–336. 10.1046/j.1472-765X.2003.01320.x 12680948

[B18] Garneau J. E. Dupuis M. È. Villion M. Romero D. A. Barrangou R. Boyaval P.. (2010). The CRISPR/Cas bacterial immune system cleaves bacteriophage and plasmid DNA. Nature 468, 67–71. 10.1038/nature09523 21048762

[B19] Garneau J. R. Depardieu F. Fortier L. C. Bikard D. Monot M. (2017). PhageTerm: a tool for fast and accurate determination of phage termini and packaging mechanism using next-generation sequencing data. Sci. Rep. 7, 8292. 10.1038/s41598-017-07910-5 28811656 PMC5557969

[B20] Goldfarb T. Sberro H. Weinstock E. Cohen O. Doron S. Charpak-Amikam Y. (2015). BREX is a novel phage resistance system widespread in microbial genomes. EMBO J. 34, 169–183. 10.15252/embj.201489455 25452498 PMC4337064

[B21] Gorzynski J. E. Goenka S. D. Shafin K. Jensen T. D. Fisk D. G. Grove M. E.. (2022). Ultrarapid nanopore genome sequencing in a critical care setting. N. Engl. J. Med. 386, 700–702. 10.1056/NEJMc2112090 35020984

[B22] Guglielmotti D. M. Mercanti D. J. Reinheimer J. A. Quiberoni A. D. L. (2011). Review: efficiency of physical and chemical treatments on the inactivation of dairy bacteriophages. Front. Microbiol. 2, 282. 10.3389/fmicb.2011.00282 22275912 PMC3257867

[B23] Hampton H. G. Watson B. N. J. Fineran P. C. (2020). The arms race between bacteria and their phage foes. Nature 577, 327–336. 10.1038/s41586-019-1894-8 31942051

[B24] Han K. Dong Y. An X. Song L. Li M. Fan H.. (2022). Potential application of a newly isolated phage BUCT609 infecting *Stenotrophomonas maltophilia*. Front. Microbiol. 13, 1001237. 10.3389/fmicb.2022.1001237 36478859 PMC9720304

[B25] Han P. Niestemski L. R. Barrick J. E. Deem M. W. (2013). Physical model of the immune response of bacteria against bacteriophage through the adaptive CRISPR-Cas immune system. Phys. Biol. 10, 025004. 10.1088/1478-3975/10/2/025004 23492852 PMC3652287

[B26] Hernandez A. J. Lee S. J. Thompson N. J. Griffith J. D. Richardson C. C. (2022). Residues located in the primase domain of the bacteriophage T7 primase-helicase are essential for loading the hexameric complex onto DNA. J. Biol. Chem. 298, 101996. 10.1016/j.jbc.2022.101996 35500649 PMC9198812

[B27] Hernandez A. J. Richardson C. C. (2019). Gp2.5, the multifunctional bacteriophage T7 single-stranded DNA binding protein. Semin. Cell Dev. Biol. 86, 92–101. 10.1016/j.semcdb.2018.03.018 29588157 PMC6162179

[B28] Hryhorowicz M. Lipiński D. Zeyland J. Słomski R. (2017). CRISPR/Cas9 immune system as a tool for genome engineering. Arch. Immunol. Ther. Exp. 65, 233–240. 10.1007/s00005-016-0427-5 27699445 PMC5434172

[B29] Hynes A. P. Labrie S. J. Moineau S. (2016). Programming native CRISPR arrays for the generation of targeted immunity. mBio 7, 16. 10.1128/mBio.00202-16 27143383 PMC4959665

[B30] Jakutyte-Giraitiene L. Gasiunas G. (2016). Design of a CRISPR-Cas system to increase resistance of Bacillus subtilis to bacteriophage SPP1. J. Ind. Microbiol. Biotechnol. 43, 1183–1188. 10.1007/s10295-016-1784-0 27255973

[B31] Kim S. Jeong H. Kim E. Y. Kim J. F. Lee S. Y. Yoon S. H.. (2017). Genomic and transcriptomic landscape of *Escherichia coli* BL21(DE3). Nucleic Acids Res. 45, 5285–5293. 10.1093/nar/gkx228 28379538 PMC5435950

[B32] Kongari R. Rajaure M. Cahill J. Rasche E. Mijalis E. Berry J.. (2018). Phage spanins: diversity, topological dynamics and gene convergence. BMC Bioinformatics 19, 326. 10.1186/s12859-018-2342-8 30219026 PMC6139136

[B33] Labrie S. J. Samson J. E. Moineau S. (2010). Bacteriophage resistance mechanisms. Nat. Rev. Microbiol. 8, 317–327. 10.1038/nrmicro2315 20348932

[B34] LeRoux M. Laub M. T. (2022). Toxin-antitoxin systems as phage defense elements. Annu. Rev. Microbiol. 76, 21–43. 10.1146/annurev-micro-020722-013730 35395167

[B35] Li P. Lin H. Mi Z. Xing S. Tong Y. Wang J.. (2019). Screening of polyvalent phage-resistant *Escherichia coli* strains based on phage receptor analysis. Front. Microbiol. 10, 850. 10.3389/fmicb.2019.00850 31105661 PMC6499177

[B36] Loenen W. A. Dryden D. T. Raleigh E. A. Wilson G. G. Murray N. E. (2014). Highlights of the DNA cutters: a short history of the restriction enzymes. Nucleic Acids Res 42, 3–19. 10.1093/nar/gkt990 24141096 PMC3874209

[B37] Lopatina A. Tal N. Sorek R. (2020). Abortive infection: bacterial suicide as an antiviral immune strategy. Annu. Rev. Virol. 7, 371–384. 10.1146/annurev-virology-011620-040628 32559405

[B38] Los M. (2012). Minimization and prevention of phage infections in bioprocesses. Methods Mol. Biol. 834, 305–315. 10.1007/978-1-61779-483-4_19 22144367

[B39] Lu B. Yao X. Han G. Luo Z. Zhang J. Yong K.. (2022). Isolation of Klebsiella pneumoniae Phage vB_KpnS_MK54 and pathological assessment of endolysin in the treatment of pneumonia mice model. Front. Microbiol. 13, 854908. 10.3389/fmicb.2022.854908 35387089 PMC8978833

[B40] Ma Y. Zhang L. Huang X. (2014). Genome modification by CRISPR/Cas9. FEBS J. 281, 5186–5193. 10.1111/febs.13110 25315507

[B41] Makarova K. S. Koonin E. V. (2015). Annotation and classification of CRISPR-cas systems. Methods Mol. Biol. 1311, 47–75. 10.1007/978-1-4939-2687-9_4 25981466 PMC5901762

[B42] Makarova K. S. Wolf Y. I. Koonin E. V. (2013). Comparative genomics of defense systems in archaea and bacteria. Nucleic Acids Res. 41, 4360–4377. 10.1093/nar/gkt157 23470997 PMC3632139

[B43] Meier-Kolthoff J. P. Goker M. (2017). VICTOR: genome-based phylogeny and classification of prokaryotic viruses. Bioinformatics 33, 3396–3404. 10.1093/bioinformatics/btx440 29036289 PMC5860169

[B44] Moraru C. Varsani A. Kropinski A. M. (2020). VIRIDIC-a novel tool to calculate the intergenomic similarities of prokaryote-infecting viruses. Viruses 12, 268. 10.3390/v12111268 33172115 PMC7694805

[B45] Nandi S. Whitby M. C. (2012). The ATPase activity of Fml1 is essential for its roles in homologous recombination and DNA repair. Nucleic Acids Res. 40, 9584–9595. 10.1093/nar/gks715 22844101 PMC3479183

[B46] Nielsen J. Keasling J. D. (2016). Engineering cellular metabolism. Cell 164, 1185–1197. 10.1016/j.cell.2016.02.004 26967285

[B47] Ofir G. Melamed S. Sberro H. Mukamel Z. Silverman S. Yaakov G.. (2018). DISARM is a widespread bacterial defence system with broad anti-phage activities. Nat. Microbiol. 3, 90–98. 10.1038/s41564-017-0051-0 29085076 PMC5739279

[B48] Overbeek R. Olson R. Pusch G. D. Olsen G. J. Davis J. J. Disz T.. (2014). The SEED and the rapid annotation of microbial genomes using subsystems technology (RAST). Nucleic Acids Res. 42, D206–D214. 10.1093/nar/gkt1226 24293654 PMC3965101

[B49] Prevelige Jr P. E. Cortines J. R. (2018). Phage assembly and the special role of the portal protein. Curr. Opin. Virol. 31, 66–73. 10.1016/j.coviro.2018.09.004 30274853

[B50] Prjibelski A. Antipov D. Meleshko D. Lapidus A. Korobeynikov A. (2020). Using SPAdes de novo assembler. Curr. Protoc. Bioinf. 70, e102. 10.1002/cpbi.102 32559359

[B51] Ray K. Oram M. Ma J. Black L. W. (2009). Portal control of viral prohead expansion and DNA packaging. Virology 391, 44–50. 10.1016/j.virol.2009.05.029 19541336 PMC2739799

[B52] Roberts R. J. Belfort M. Bestor T. Bhagwat A. S. Bickle T. A. Bitinaite J.. (2003). A nomenclature for restriction enzymes, DNA methyltransferases, homing endonucleases and their genes. Nucleic Acids Res. 31, 1805–1812. 10.1093/nar/gkg274 12654995 PMC152790

[B53] Rocha E. P. C. Bikard D. (2022). Microbial defenses against mobile genetic elements and viruses: who defends whom from what? PLoS Biol. 20, e3001514. 10.1371/journal.pbio.3001514 35025885 PMC8791490

[B54] Safari F. Sharifi M. Farajnia S. Akbari B. Karimi Baba Ahmadi M. Negahdaripour M.. (2020). The interaction of phages and bacteria: the co-evolutionary arms race. Crit. Rev. Biotechnol. 40, 119–137. 10.1080/07388551.2019.1674774 31793351

[B55] Santos C. N. Regitsky D. D. Yoshikuni Y. (2013). Implementation of stable and complex biological systems through recombinase-assisted genome engineering. Nat. Commun. 4, 2503. 10.1038/ncomms3503 24056574

[B56] Shaer Tamar E. Kishony R. (2022). Multistep diversification in spatiotemporal bacterial-phage coevolution. Nat. Commun. 13, 7971. 10.1038/s41467-022-35351-w 36577749 PMC9797572

[B57] Stoddard B. L. (2005). Homing endonuclease structure and function. Q. Rev. Biophys. 38, 49–95. 10.1017/S0033583505004063 16336743

[B58] Sullivan M. J. Petty N. K. Beatson S. A. (2011). Easyfig: a genome comparison visualizer. Bioinformatics 27, 1009–1010. 10.1093/bioinformatics/btr039 21278367 PMC3065679

[B59] Summers D. K. Beton C. W. Withers H. L. (1993). Multicopy plasmid instability: the dimer catastrophe hypothesis. Mol. Microbiol. 8, 1031–1038. 10.1111/j.1365-2958.1993.tb01648.x 8361350

[B60] Taslem Mourosi J. Awe A. Guo W. Batra H. Ganesh H. Wu X.. (2022). Understanding bacteriophage tail fiber interaction with host surface receptor: the key “blueprint” for reprogramming phage host range. Int. J. Mol. Sci. 23, 1–14. 10.3390/ijms232012146 36292999 PMC9603124

[B61] van Houte S. Buckling A. Westra E. R. (2016). Evolutionary ecology of prokaryotic immune mechanisms. Microbiol. Mol. Biol. Rev. 80, 745–763. 10.1128/MMBR.00011-16 27412881 PMC4981670

[B62] Walker S. A. Klaenhammer T. R. (2000). An explosive antisense RNA strategy for inhibition of a lactococcal bacteriophage. Appl. Environ. Microbiol. 66, 310–319. 10.1128/AEM.66.1.310-319.2000 10618241 PMC91823

[B63] Wick R. R. Schultz M. B. Zobel J. Holt K. E. (2015). Bandage: interactive visualization of de novo genome assemblies. Bioinformatics 31, 3350–3352. 10.1093/bioinformatics/btv383 26099265 PMC4595904

[B64] Xu J. Hendrix R. W. Duda R. L. (2014). Chaperone-protein interactions that mediate assembly of the bacteriophage lambda tail to the correct length. J. Mol. Biol. 426, 1004–1018. 10.1016/j.jmb.2013.06.040 23911548 PMC3907469

[B65] Yuen K. S. Chan C. P. Wong N. H. M. Ho C. H. Ho T. H. Lei T.. (2015). CRISPR/Cas9-mediated genome editing of Epstein-Barr virus in human cells. J. Gen. Virol. 96, 626–636. 10.1099/jgv.0.000012 25502645

[B66] Zhao L. Kanamaru S. Chaidirek C. C. Arisaka F. (2003). 15 and P3, the tail completion proteins of bacteriophage T4, both form hexameric rings. J. Bacteriol. 185, 1693–1700. 10.1128/JB.185.5.1693-1700.2003 12591887 PMC148078

[B67] Zou X. Xiao X. Mo Z. Ge Y. Jiang X. Huang R.. (2022). Systematic strategies for developing phage resistant *Escherichia coli* strains. Nat. Commun. 13, 4491. 10.1038/s41467-022-31934-9 35918338 PMC9345386

