# Supplementary material for: Characterization, complete genome sequencing, and CRISPR/Cas9 system-based decontamination of a novel Escherichia coli phage TR1 from fermentation substrates
Source: Front Microbiol. 2023 Aug 11;14:1230775. doi: 10.3389/fmicb.2023.1230775 (PMC10450929; doi:10.3389/fmicb.2023.1230775)
Supplement: Supplementary file 1 [file Data_Sheet_1.docx]

# Supporting information for

# Characterization, complete genome and CRISPR/Cas9 system-based decontamination of a novel *Escherichia coli* phage TR1 from fermentation substrates

Yuqi Dong^a^, Yunfei Huang^a^, Huahao Fan^a^, Lihua Song^a^, Xiaoping An^a^, Shan Xu^a^, Mengzhe Li^a,^*, Yigang Tong^a,b,^*

a College of Life Science and Technology, Beijing University of Chemical Technology, Beijing 100029, China;

b Beijing Advanced Innovation Center for Soft Matter Science and Engineering, Beijing University of Chemical Technology, Beijing 100029, China;

Yuqi Dong and Yunfei Huang are contributed equally to this work.

***Correspondence:**

Yigang Tong:

[tong.yigang@gmail.com](mailto:tong.yigang@gmail.com)

Mengzhe Li:

futurelmz123@163.com

Table S1. Phages homologous to phage TR1 in GenBank

| **Scientific Name** | **Cover** | **E-value** | **Identity (%)** | **Length (bp)** | **Accession number** |
| --- | --- | --- | --- | --- | --- |
| *Escherichia* phage TR1 | 100% | 0 | 100.00 | 44099 | OP087522.1 |
| *Escherichia* phage vB_EcoS_IME542 | 68% | 0 | 99.70 | 46553 | NC_048208.1 |
| *Escherichia* phage vB_EcoS_CEB_EC3a | 59% | 0 | 84.59 | 44234 | NC_047812.1 |
| *Escherichia* phage JeanPiccard | 63% | 0 | 85.20 | 47149 | MZ501080.1 |
| *Escherichia* phage vB_EcoS-2862V | 64% | 0 | 85.22 | 44219 | MK907276.1 |
| *Escherichia* phage vB_EcoS-2862I | 62% | 0 | 85.20 | 44219 | MK907272.1 |
| *Escherichia* phage vB_EcoS-26174II | 65% | 0 | 85.20 | 44219 | MK907264.1 |
| *Escherichia* phage vB_EcoS-26046IV | 61% | 0 | 85.20 | 44219 | MK907259.1 |
| *Escherichia* phage vB_EcoS-25988I | 64% | 0 | 85.20 | 44219 | MK907248.1 |
| *Escherichia* phage vB_EcoS-2006V | 63% | 0 | 85.22 | 44219 | MK907247.1 |
| *Escherichia* phage vB_EcoS_FP | 68% | 0 | 85.15 | 43757 | MT682706.1 |
| *Escherichia* phage 2725-N35 | 54% | 0 | 84.12 | 45917 | MN840485.1 |
| *Escherichia* phage vB_EcoS-IME253 | 46% | 0 | 94.12 | 46717 | NC_047810.1 |
| *Escherichia* phage ZL19 | 43% | 0 | 93.88 | 47884 | OM258170.1 |
| *Escherichia* phage vB_EcoS_MM01 | 51% | 0 | 85.27 | 43157 | MK373793.1 |
| *Escherichia* phage vB_EcoS_ACG-M12 | 55% | 0 | 84.52 | 46054 | JN986845.1 |
| *Escherichia* phage AugustePiccard | 59% | 0 | 84.62 | 50126 | MZ501051.1 |
| *Escherichia* phage DTL | 58% | 0 | 83.87 | 45814 | NC_047893.1 |
| *Escherichia* phage vB_EcoS_SCS31 | 55% | 0 | 84.32 | 46054 | ON081052.1 |
| *Escherichia* phage vB_EcoS-2006IV | 65% | 0 | 85.47 | 44219 | MK907246.1 |
| *Escherichia* phage JulesPiccard | 54% | 0 | 86.09 | 47731 | MZ501087.1 |
| *Escherichia* phage vB_EcoD_SU57 | 64% | 0 | 84.00 | 46150 | MT511058.1 |
| *Escherichia* phage Rtp | 58% | 0 | 85.41 | 46219 | NC_007603.1 |
| *Escherichia* phage vB_EcoS-26020III | 65% | 0 | 86.00 | 44219 | MK907253.1 |
| *Escherichia* phage CJ19 | 9% | 0 | 87.56 | 49567 | MT176427.1 |
| *Escherichia* phage phiEB49 | 22% | 0 | 76.74 | 47180 | JF770475.1 |
| *Escherichia* virus ECH1 | 37% | 0 | 87.39 | 49553 | MN164484.1 |
| *Escherichia* phage IMM-001 | 40% | 0 | 75.31 | 32486 | MF630922.1 |
| *Escherichia* phage e4/1c | 19% | 0 | 74.08 | 47112 | KJ668713.1 |
| *Escherichia* phage vB_EcoS_AHS24 | 15% | 0 | 74.09 | 46440 | KF771238.1 |
| *Escherichia* phage Jk06 | 16% | 0 | 73.51 | 46072 | DQ121662.1 |
| *Escherichia* phage phiJLA23 | 16% | 0 | 73.43 | 43017 | NC_047740.1 |
| *Escherichia* phage phiKP26 | 15% | 0 | 73.77 | 47285 | NC_042038.1 |
| *Escherichia* phage C119 | 15% | 0 | 73.76 | 47319 | NC_042046.1 |
| *Escherichia* phage vB_EcoS_AKS96 | 16% | 0 | 73.32 | 45746 | KF771239.1 |
| *Escherichia* phage vB_EcoS-BECP10 | 21% | 0 | 73.57 | 47915 | MW286156.1 |
| *Escherichia* phage vB_EcoS_Rogue1 | 16% | 0 | 73.37 | 45805 | JQ182736.1 |
| *Escherichia* phage vB_EcoS_AHP42 | 13% | 0 | 73.87 | 46847 | KF771237.1 |
| *Escherichia* phage vB_EcoD_Opt-719 | 13% | 0 | 74.68 | 48302 | OL539451.1 |
| *Shigella* phage Sf12 | 11% | 0 | 73.87 | 47647 | NC_047848.1 |
| *Escherichia* phage P818 | 15% | 0 | 76.52 | 49175 | ON062054.1 |
| *Shigella* phage Sd1 | 21% | 0 | 75.97 | 48262 | NC_047847.1 |
| *Escherichia* phage vB_EcoS_ESCO41 | 16% | 0 | 80.74 | 50800 | NC_047820.1 |
| *Escherichia* phage P817 | 6% | 0 | 76.42 | 50751 | MZ826699.1 |
| phage vB_EcoM-p111 | 8% | 0 | 79.55 | 50241 | OL449681.1 |

Table S2. Eight different treatment groups in anti-phage activities of recombinant strains assay

| Group | Strain name | Gentamicin^1^ | Arabinlose^2^ | Phage TR1 infection^3^ |
| --- | --- | --- | --- | --- |
| A | BL21-C | + | - | - |
| B | BL21-C | + | - | + |
| C | BL21-C | + | + | + |
| D | BL21-T | + | - | - |
| E | BL21-T | + | - | + |
| F | BL21-T | + | + | + |
| G | BL21(DE3) | NA | NA | + |
| H | BL21(DE3) | NA | NA | - |

1. The final concentration of gentamicin was 20 μg/mL in LB broth.

2. The final concentration of arabinose was 0.2% in LB broth.

3. Phage infection groups were challenged with a titer of 5*107 PFU/mL of phage TR1.
